# Neuroprotection and Enhanced Learning and Memory Abilities of Steamed American Ginseng (*Panax quinquefolium* L.) Based on Zebrafish

**DOI:** 10.1002/fsn3.70369

**Published:** 2025-06-01

**Authors:** Yuting Yang, Shuyun Liang, Mengdan Xu, Xiaokang Liu, Yunlong Guo, Jiyu Gong, Liru Zhao, Guangzhi Cai

**Affiliations:** ^1^ School of Pharmaceutical Sciences Changchun University of Chinese Medicine Changchun China; ^2^ The First People's Hospital of Zhengzhou Zhengzhou China; ^3^ National Engineering Research Center of TCM Standardization Technology, Shanghai Institute of Materia Medica Chinese Academy of Sciences Shanghai China; ^4^ School of Chinese Materia Medica Nanjing University of Nature Herbs Nanjing China; ^5^ Zhongshan Institute for Drug Discovery, Shanghai Institute of Materia Medica Chinese Academy of Sciences Zhongshan China; ^6^ Jilin Ginseng Academy Changchun University of Chinese Medicine Changchun China; ^7^ Jilin Institute of Biology Changchun China

**Keywords:** learning and memory, neuroprotection, *Panax quinquefolius* L., steaming, total saponins, zebrafish

## Abstract

American ginseng ( *Panax quinquefolius* L.) exhibits multiple pharmacological properties, including anti‐inflammatory, antioxidant, and neuroprotective effects. In 2023, it was officially approved as a dual‐purpose substance for both medicinal and food applications, establishing it as a scientifically validated ingredient for functional foods and health supplements. We extracted total saponins (AGTS0‐AGTS9) from American ginseng subjected to different steaming‐drying cycles to explore how the steaming process affects saponin composition and neuroprotective activity. Using ultra‐high‐performance liquid chromatography coupled with quadrupole Orbitrap mass spectrometry (UHPLC‐Q‐Orbitrap/MS) technology, 35 types of ginsenosides were identified from AGTS0‐AGTS9. With increasing steaming cycles, the original ginsenoside content decreased, while the rare ginsenoside content gradually increased. During steaming, ginsenosides undergo deglycosylation, hydrolysis, and acetylation, transforming into rare ginsenosides. We employed two models–neuronal injury in transgenic zebrafish larvae and cognitive impairment in wild‐type AB adults, both induced by AlCl_3_–to assess the neuroprotective effects of AGTS0‐AGTS9 and the memory‐enhancing potential of AGTS5, respectively. Results showed that AGTS4, AGTS5, and AGTS6 markedly increased the neuronal fluorescence area and intensity in larvae, reduced acetylcholinesterase (AChE), malondialdehyde (MDA), tumor necrosis factor‐α (TNF‐α), and interleukin‐6 (IL‐6) levels in larval tissues, while elevating acetylcholine (ACh), superoxide dismutase (SOD), glutathione peroxidase (GSH‐Px), and catalase (CAT) activities. AGTS5 exhibited the most potent neuroprotective activity. In adult zebrafish, AGTS5 significantly improved locomotor performance in the Novel Tank Test (NTT) and T‐maze, increased Nissl body counts in the brain, reduced AChE activity and MDA/TNF‐α/IL‐6 levels, and enhanced ACh, SOD, GSH‐Px, and CAT content. These findings demonstrate that AGTS5 holds promise as a functional food or nutritional supplement for neuroprotection and cognitive enhancement.

## Graphical abstract

Our research is the first to explore the neuroprotective effects of total saponin active compounds isolated from *Panax quinquefolius* after steaming different times, and to screen out those total saponins with better neuroprotective activity, as well as to conduct a preliminary study on their potential to improve learning and memory impairments. Results indicate that total saponins extracted from steaming American ginseng processed by five steaming and five drying(AGTS5) exhibit good neuroprotective effects. Furthermore, our experiments have also demonstrated that AGTS5 can exert neuroprotective effects and improve learning and memory by increasing Nissl body counts in the brain, reducing oxidative stress, decreasing inflammation, and inhibiting cholinesterase activity.

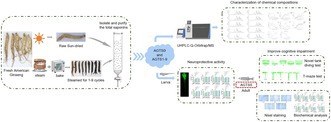

## 1 Introduction

*Panax quinquefolium*, commonly known as American ginseng, is a prized herb extensively used in pharmaceuticals, nutritional supplements, and culinary items. As a kind of medicine‐food homology substance, American ginseng not only possesses nutritional value but also can prevent and treat diseases as well as strengthen the body, making it especially suitable for elderly and frail individuals. Ginsenoside, the active ingredient of American ginseng, demonstrates a range of pharmacological activities, including anti‐inflammatory, antioxidant, antitumor, neuroprotective, and cardioprotective properties (Shan et al. 2023; Szczuka et al. 2019; Zhang et al. 2023). The steaming of American ginseng has a long history. According to the *Essentials of Materia Medica* “Recently, some people have found American ginseng to be cold in nature and have been steaming it dozens of times in a rice cooker.” Modern research has confirmed that the cooling properties of American ginseng can be reduced through steaming (Li et al. 2021). Hence, the applicable population for American ginseng has increased. Besides, studies have shown that during the steaming process, primary ginsenosides can be transformed through a series of reactions, such as hydrolysis, deglycosylation, and isomerization, into rare ginsenosides with lower polarity (Fan et al. 2024; Huang, Li, et al. 2019; Huang, Liu, et al. 2019; Sun et al. 2012). These minimal polarity ginsenosides exhibit good therapeutic effects on blood and nervous system diseases, as well as in anticancer and antitumor treatments (Shan et al. 2023; Shin et al. 2016; Wang et al. 2013). At present, researchers are mainly focused on the chemical changes of ginsenoside components in American ginseng before and after steaming, while the study on how the steaming process affects the pharmacological activities of American ginseng is still relatively scarce.

Currently, the prevalence rate of neurodegenerative diseases in society is increasing year by year. For example, Alzheimer's disease (AD), a form of dementia, is characterized by neuronal damage in the brain, resulting in impaired communication between neurons (Fenton et al. 2022). This condition is marked by a progressive decline in learning and memory functions, often accompanied by anxiety and depression (Fenton et al. 2022). According to the 2024 Alzheimer's Disease Facts and Figures report, 6.9 million Americans aged 65 and above are currently living with AD, and this figure is projected to escalate to 13.8 million by 2060, posing a tremendous burden on patients' families and society (Jia et al. 2018). Therefore, it is crucial to identify safe and effective medications that can slow down the progression of AD. Extracts obtained from natural plants have great potential, such as Hericium erinaceus (Bull.) Pers. ethanolic extract and [6]‐Gingerol, which can significantly improve scopolamine‐induced learning and memory impairment (Kim et al. 2018; Valu et al. 2021).

As one of the animal models for studying neurodegenerative diseases, zebrafish have gained widespread popularity due to their convenient operation, clear genetic background, and high similarity to human neural networks (Bashirzade et al. 2022). Aluminum (Al) can penetrate the blood–brain barrier, gradually accumulate in brain tissue, decrease ACh levels in zebrafish brains, disrupt neuronal structure, reduce the expression of brain‐derived neurotrophic factor and mammalian rapamycin target protein genes, increase the expression of amyloid precursor protein (APP) and Tau protein, leading to cognitive impairments (Gao et al. 2022; Niu 2018). Al can also trigger neuroinflammation by activating microglia. In addition, it can disrupt the intracellular redox balance, induce oxidative stress, and further exacerbate cognitive impairment (Borai et al. 2017). Evidence has shown that long‐term exposure to AlCl_3_ can lead to cognitive impairment in zebrafish (Gao et al. 2022; Kaur et al. 2022). The HuC gene is specifically expressed in vertebrate neurons and shares homology with Drosophila's Elav (Kim et al. 1996). It can promote neuronal differentiation and maturation by regulating RNA (Perrone‐Bizzozero and Bird 2013). Meanwhile, Purkinje neurons, which are nerve cells that play a crucial role in the cerebellum, are closely related to associative learning and are considered potential therapeutic targets for neurological diseases (Chen et al. 2022). Studies have shown that the HuC gene can affect the progression of neurodegenerative diseases by maintaining the stability of Purkinje neuron dendrites (Ogawa et al. 2018). The transgenic Tg (HuC: EGFP) lines allow the HuC protein in the brains of zebrafish to be labeled with green fluorescence, which can be used to directly reflect neuronal development (Choe et al. 2021). This is often applied to observe the effects of drugs on the nervous system (Liang et al. 2023; Wang et al. 2022).

In order to gain a better understanding of the impact of the number of steaming and baking processes on the total saponins in American ginseng (American ginseng total saponins, AGTS) and its neuroprotective activity, this study systematically prepared sun‐dried American ginseng and samples of American ginseng steamed and baked one to nine times. The AGTS was then extracted and isolated from these samples. The Ultra‐high performance liquid chromatography‐quadrupole‐orbitrap high‐resolution mass spectrometry (UHPLC‐Q‐Orbitrap/MS) technology was adopted to characterize and analyze the AGTS. Meanwhile, using the zebrafish model, the potential neuroprotective effect of AGTS was evaluated by assessing its intervention on nerve damage and cognitive impairment induced by aluminium trichloride (AlCl_3_). This clarified the potential neuroprotective effect of AGTS. This research will provide new ideas for the processing technology of American ginseng and offer scientific evidence for the development of natural and effective neuroprotectants and functional foods with the function of enhancing memory.

## 2 Materials and Methods

### 2.1 Chemicals and Reagents

Donepezil hydrochloride, DP (J15HS188725) and Imipramine hydrochloride, IMP (Z26M10H84088) were purchased from Shanghai Yuanye, with purities greater than 98%; Chromatographic‐grade methanol and Chromatographic‐grade acetonitrile were purchased from Thermo Fisher Scientific; aluminium chloride (AlCl_3_) was purchased from Xi Long Scientific (1907262); zebrafish acetylcholine, ACh (YX‐010308Z), acetylcholinesterase, AChE (YX‐010313Z), zebrafish malondialdehyde, MDA (YX‐130401Z), zebrafish superoxide dismutase, SOD (YX‐191504Z), zebrafish glutathione peroxidase, GSH‐Px (YX‐071624Z), zebrafish catalase, CAT (YX‐030120Z), zebrafish tumor necrosis factor‐α, TNF‐α (YX‐201407Z), and zebrafish interleukin‐6, IL‐6 (YX‐091260Z) assay kits were purchased from Shanghai Youxuan; Bouin's fixative was purchased from Jinclone.

### 2.2 Sample Collection and Processing

The fresh American ginseng was purchased from Tieli City, Heilongjiang Province, and identified by Associate Professor Guangzhi Cai from Changchun University of Chinese Medicine as the fresh roots of *Panax quinquefolium* L., a plant belonging to the genus Panax in the family Araliaceae. Then, fresh American ginseng was steamed at a temperature of 98°C for 3 h and subsequently dried at 60°C for 14 h. This process was repeated nine times in total, with a random sample taken after each steaming. This resulted in obtaining steamed American ginseng samples numbered from one to nine. The sun‐dried American ginseng was prepared according to the requirements under the item of American ginseng in the Chinese Pharmacopeia (2020 Edition) and was recorded as S0. These samples were ground, extracted ultrasonically with 70% ethanol for 30 min, filtered, and the solvent from the filtrate was recovered for further use.

### 2.3 Preparation of AGTS Extracts

The D101 resin was selected as an ideal adsorbent due to its favorable adsorption and desorption properties. It was pretreated and used for the separation and purification of total saponins (Liu et al. 2019). A sample solution (0–9), prepared as described above, underwent dynamic adsorption at a flow rate of 1 mL/min, followed by static adsorption for 4 h. Distilled water was first used to elute sugars and impurities, after which 70% ethanol was employed to elute the saponins until no saponin reaction was detected (Lin et al. 2022). The eluent was collected and freeze‐dried using a vacuum freeze dryer to obtain the total saponins from American ginseng steamed 0–9 times, labeled as AGTS0‐AGTS9.

### 2.4 Chemical Composition Analysis of AGTS0‐AGTS9

The AGTS0‐AGTS9 lyophilized powders were precisely weighed, dissolved in chromatography‐grade methanol to prepare a 3 mg/mL solution, filtered through a 0.22 μm membrane, and then subjected to UHPLC‐Q‐Orbitrap/MS analysis. A reversed‐phase C_18_ column (150 × 4.6 mm, 2.7 μm, Agilent) was used for the separation of BAGTS. The mobile phase consisted of 0.1% formic acid (solvent A) and acetonitrile (solvent B), with a gradient elution program as follows: 0–5 min, 18%–20% B; 5–7 min, 20%–30% B; 7–10 min, 30%–32% B; 10–12 min, 32%–33% B; 12–16 min, 33%–75% B; 16–20 min, 18% B. The column temperature was maintained at 25°C, with a flow rate of 0.4 mL/min. The injection volume was 5 μL.

The Q‐Exactive Orbitrap mass spectrometer (manufactured by Thermo Fisher Scientific, USA) was operated with an ESI ion source in negative ion mode with a spray voltage of −3.5 kV. The mass scanning range was set from *m*/*z* 100 to 1500. The sheath gas flow rate was 35 Arb, the auxiliary gas flow rate was 10 Arb, the S‐Lens RF level was adjusted to 55%, and the capillary temperature was maintained at 320°C.

### 2.5 Zebrafish Maintenance and Embryo Harvesting

In this study, we utilized two zebrafish strains: the transgenic Tg(HuC:EGFP) and the wild‐type AB line, both obtained from Changchun University of Chinese Medicine. The fish were maintained under a 14 h/10 h light/dark cycle at 28°C ± 0.5°C and fed brine shrimp twice daily. For embryo collection, Tg(HuC:EGFP) males and females were placed in a spawning tank at a 1:1 ratio, separated by a partition. The next morning, the partition was removed, and light exposure stimulated natural spawning. Fertilized eggs were then collected and cultured in E3 medium.

### 2.6 Neuroprotective Activity Profiling of AGTS0‐AGTS9

With slight modifications based on the referenced literature method (Wang et al. 2022; Zhong et al. 2022). Well‐developed Tg(HuC:EGFP) zebrafish embryos at 3 days postfertilization (3 dpf) were placed into 6‐well plates. They were randomly assigned to 12 groups: a control group (culture medium), a model group (0.1 μg/mL AlCl_3_ solution), and AGTS0‐AGTS9 groups (1 μg/mL + 0.1 μg/mL AlCl_3_). There were 30 embryos per well, and three parallel wells per group. Subsequently, the larvae were incubated in a 28°C incubator until 72 h. After incubation, the larvae were anesthetized with 3‐Ethoxycarbonylaniline mesylate and then fixed with 5% sodium carboxymethyl cellulose. Finally, images were captured on an inverted fluorescence microscope. The exposure time was set at 1 s and 139.376 ms, and the gain was set at 2720%. The Image J software was used to analyze the fluorescence area and intensity of zebrafish neurons in each group.

Zebrafish larvae from each group (three parallel samples per group, 30 larvae per sample) were collected in centrifuge tubes. After complete removal of the drug solution, the larvae were gently rinsed three times with physiological saline. Following the final rinse, residual liquid was thoroughly aspirated and the larvae were weighed. An appropriate amount of normal saline was added as instructed in the kit manual, then the samples were homogenized in an ice‐water bath. The homogenate was then centrifuged at 13,000 × *g* for 10 min. The supernatant was collected, aliquoted, and stored at −20°C for subsequent determination of acetylcholine (ACh), acetylcholinesterase (AChE), malondialdehyde (MDA), superoxide dismutase (SOD), catalase (CAT), glutathione peroxidase (GSH‐Px), tumor necrosis factor‐α (TNF‐α), and interleukin‐6 (IL‐6).

### 2.7 Analysis of AGTS5's Learning and Memory‐Enhancing Activity

In this study, 66 adult zebrafish (5–6 months old), including males and females, were used, and each group consisted of 11 individuals. The zebrafish were randomly allocated into five groups: a control group, an AlCl_3_ group (100 μg/L), two treatment groups of AGTS5 (0.5 and 1.5 mg/L), and two positive control groups with DP (1 mg/L) and IMP (1 mg/L). Except for the control group, all other groups were continuously soaked with AlCl_3_ for 30 days. During this period, dilute hydrochloric acid was used to adjust the pH of the solution to 6.4. Subsequently, a 14‐day therapeutic intervention was conducted, where each experimental group received their respective treatments (Gao et al. 2022). To maintain consistent drug concentrations, half of the fresh drug solution was replaced daily per group. The whole animal experiments were approved by the Animal Ethics Committee of Changchun University of Chinese Medicine (no. 2024300).

#### 2.7.1 Novel Tank Test (NTT)

The NTT is one of the most widely used experimental procedures for accessing the upward movement and exploratory behavior of zebrafish. It can be utilized to observe zebrafish's anxiety responses to a new environment. This usage follows the method outlined by (Valu et al. 2021). When the administration of the medication was completed, zebrafish were used for NTT testing. In the trial, zebrafish were individually placed in a 1.5 L trapezoidal tank (15.12 × 8.9 × 23.9 × 7.4 cm), and their behavior trajectories were recorded for 6 min. Tox Trac software was used to analyze the latency to reach the top of the tank, the time spent at the top, the time spent at the bottom, and the total distance moved. IMP (1 mg/L) was taken as the positive medicine in the NTT test.

#### 2.7.2 T‐Maze Test

The T‐maze is a classical experiment for detecting the learning and memory ability of zebrafish, capitalizing on their innate foraging and exploratory tendencies. This approach provides high accuracy in assessing long‐term memory formation, making it particularly valuable in neurobehavioral research. In this study, the T‐maze experiment was carried out with some minor modifications to the methods described in the literature (Pilehvar et al. 2020). The behavioral tests were conducted using a custom‐built T‐maze fabricated from clear polymethyl methacrylate, featuring two identical short arms (20 × 10 × 10 cm) and a single long arm (40 × 10 × 10 cm). The left short arm contained a green‐marked sleeve designated as the Exploration‐Choice (EC) area for behavioral tracking. All trials were executed between 8:00 am and 2:00 pm. Before the experiment began, zebrafish that were given diverse therapies were allowed to move freely in a T‐maze without red and green color covers for 1 h. During each training period, the zebrafish were transferred into the start box for 1 min individually. Afterwards, the mobile spacer was opened to let the fish swim in the T‐maze. Each fish was trained once a day for 4 consecutive days. Zebrafish that found the EC zone within 6 min were rewarded with harvested shrimp. If they did not, they were guided to the EC area and remained there for 1 min. On the fifth day, which was the trial stage, there were no harvested shrimps in the EC zone. Therefore, zebrafish would stay and search for food based on their memory from the previous 4 days. The behavior of zebrafish within 6 min, including the latency to reach the EC area and the number of entries into the EC zone in the T‐maze, was recorded by a camera. DP (1 mg/L) was taken as the positive medicine in this test.

#### 2.7.3 Nissl Staining

After the behavioral experiment, three zebrafish were randomly selected from each group. Their brain tissues were rapidly removed on ice and fixed in Bouin's fixative for 12 h. Subsequently, the tissues underwent paraffin embedding and slicing. The slices were then dewaxed with xylene, hydrated through a gradient of ethanol, and stained with toluidine blue for 5 min. Differentiation was performed with 1% glacial acetic acid, followed by rinsing with water to stop the staining process. The slides were air‐dried and cleared in xylene for 5 min before being mounted with neutral gum. Finally, images were captured under a microscope. The number of Nissl bodies in the brains of zebrafish in each group was counted using Image J software.

#### 2.7.4 Biochemical Analysis

After behavioral tests, zebrafish were euthanized in ice water (2°C–4°C) for 10 min until the opercular movement ceased (Batista et al. 2018). Subsequently, the brain tissue was quickly removed on ice. Saline was then added according to the kit instructions, and the samples were homogenized in an ice‐water bath. The homogenate was centrifuged at 13,000 × *g* for 10 min. The supernatant was collected, aliquoted, and stored at −20°C for subsequent determination of ACh, AChE, MDA, SOD, CAT, GSH‐Px, TNF‐α, and IL‐6.

### 2.8 Data Analysis

Statistical analysis of the data was conducted using GraphPad Prism 9.5.1. All data were presented as mean ± SD. Prior to analysis, the data underwent tests for both normality and homogeneity of variance. For data adhering to a normal distribution, one‐way ANOVA was applied, whereas the Kruskal–Wallis test was utilized for data that deviated from normality to determine statistical significance. *p* < 0.05 was indicative of statistical significance.

## 3 Results

### 3.1 Identification of Chemical Components From AGTS0‐AGTS9

**FIGURE 1:**
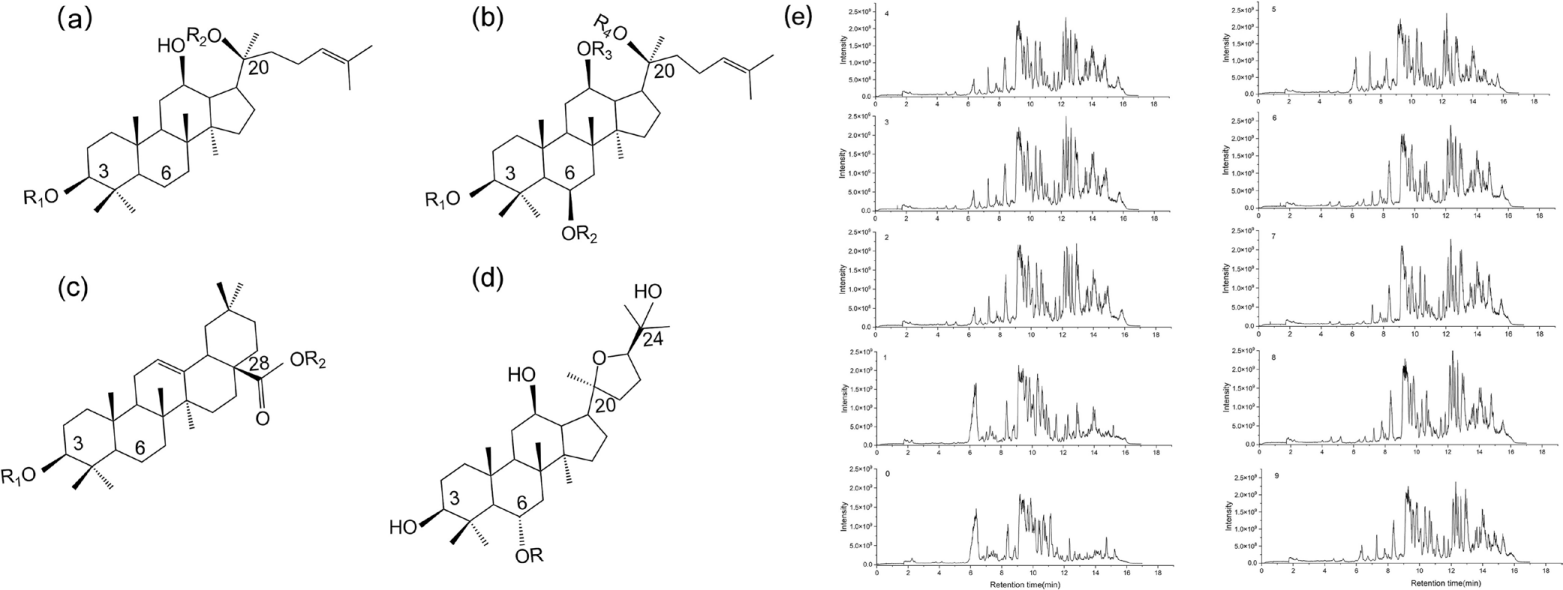
The main types of ginsenosides and the total ion chromatogram of AGTS0‐AGTS9 under negative ion mode by UHPLC‐Q‐Orbitrap/MS: (a) Protopanaxadiol saponins, (b) protopanaxatriol saponins, (c) oleanane saponins, (d) ocotillol saponins, (e) the TIC diagram of AGTS0–AGTS9.

**FIGURE 2:**
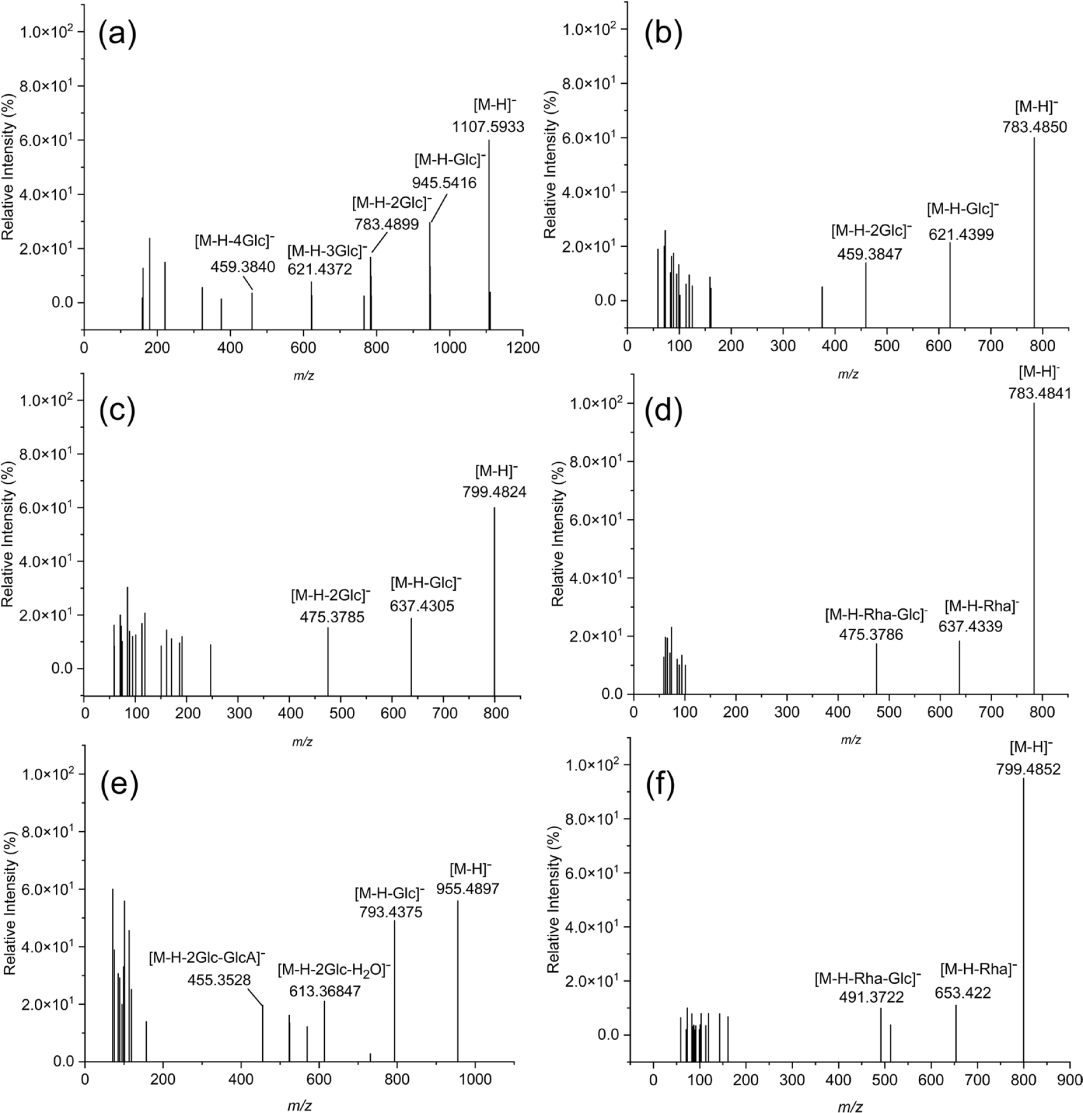
Identification of ginsenosides by UHPLC‐Q‐Orbitrap/MS in the negative ion mode: (a) Ginsenoside Rb_1_. (b) Ginsenoside Rg_3_. (c) Ginsenoside Rg_1_. (d) Ginsenoside Rg_2_. (e) Ginsenoside Ro. (f) Pseudoginsenoside F_11_.

Protopanaxadiol saponins (PPD), Protopanaxatriol saponins (PPT), Oleanolic Acid‐type saponins (OA) and Ocotillol‐type saponins (OT) are the most common types of American ginseng. The exact structures have been visualized in Figure 1a–d for clarity. The total ion chromatogram of AGTS0‐AGTS9 analyzed by UHPLC‐Q‐Orbitrap/MS is shown in Figure 1e. By comparing the accurate relative molecular masses with those of reported known ginsenosides, predicting the fragment ions potentially generated from glycosidic bond cleavage based on saponin structures, and cross‐referencing these predictions with the secondary mass spectrometry data, a total of 35 ginsenoside compounds were successfully identified from AGTS0 to AGTS9: 12 PPD‐type ginsenosides, 11 PPT‐type ginsenosides, 3 OA‐type ginsenosides, 1 OT‐type ginsenoside, and 8 other categories of ginsenosides. The detailed information of ginsenoside names, molecular formulas, and other relevant data is presented in Table 1. In negative ion mode mass spectrometry, ginsenosides readily form adduct ions. The addition of formic acid reduces the pH of the mobile phase, significantly enhancing protonation of ginsenoside molecules in the mass spectrometer's ionization source. This promotes the generation of negatively charged ions, thereby improving detection sensitivity and selectivity. Such optimization enables detailed structural characterization and accurate quantitative analysis through mass spectrometry. In the structural identification of ginsenosides, the determination of glycan types can be achieved through characteristic neutral losses: *m/z* 162.05 corresponds to the loss of a glucosyl group (‐Glc), *m/z* 146.06 indicates the loss of a rhamnosyl group (‐Rha), while *m/z* 132.04 represents the loss of either an arabinosyl (‐Ara) or xylosyl (‐Xyl) group. For PPD‐type ginsenosides, taking RT 9.28 min, *m/z* 1107.596 as an example, the identification process is described in detail as follows. Its ion peak [M − H]^−^ is at *m/z* 1107.5944, indicating that its molecular formula is C_54_H_92_O_23_. Conduct an MS fragmentation analysis of the ion peak at *m/z* 1107.5944. The secondary fragment ions at *m/z* 945.5416, 783.4899, 621.4372, and 459.3840 can be observed, as shown in Figure 2a. The ion at *m/z* 945.5416 was generated from *m/z* 1107.5944 through the loss of 1Glc. Subsequent fragmentation involved the elimination of 3Glc, ultimately yielding the characteristic PPD‐type fragment ion at *m/z* 459.3840. This fragmentation pattern is consistent with the established mass spectrometric behavior of ginsenoside Rb_1_, thereby confirming its identification (Oh et al. 2024). The compound exhibited characteristic ions at *m/z* 829.4946 [M + HCOO]^−^ and *m/z* 783.4850 [M − H]^−^ in negative ion mode, as shown in Figure 2b. The tandem MS spectrum analysis revealed diagnostic fragment ions at *m/z* 621.4399 and *m/z* 459.3847, corresponding to sequential losses of 1 and 2Glc from the precursor ion *m/z* 783.4850 [M − H]^−^. Based on this fragmentation pattern and comparison with literature data (Oh et al. 2024), the compound was unequivocally identified as ginsenoside Rg_3_.

**TABLE 1:** Table ginsenosides identified in the AGTS0‐AGTS9 samples.

No.	*t*_R_/min	Identity	Formula	Theoretical (Mass/Da)	Detected *m*/*z*	Ion adducts	Mass error (ppm)	Fragment ion	Steaming cycles
1	5.64	Noto R_1_	C_47_H_80_O_18_	932.5345	931.5248	[M + HCOO]^−^	−1.29	931.5229, 799.4915, 637.4318, 475.3796, 391.2861	0,1,2,3,4,5,6,7,8,9
2	6.13	Rg_1_	C_42_H_72_O_14_	800.4922	845.4897	[M + HCOO]^−^	0.47	799.4824, 637.4305, 475.3785	0,1,2,3,4,5,6,7,8,9
3	6.36	Re	C_48_H_82_O_18_	946.5501	945.5388	[M − H]^−^	−3.07	783.4933, 637.4285, 475.3807	0,1,2,3,4,5,6,7,8,9
4	7.12	Malonyl Re	C_51_H_84_O_21_	1032.5505	1031.5421	[M − H]^−^	0.00	945.5369, 783.4851, 637.4335, 475.3788	0,1
5	7.66	Re_2_	C_48_H_82_O_19_	962.5444	1007.5423	[M + HCOO]^−^	0.20	961.5386, 799.4863, 637.4326, 475.3785	0,1,2,3,4,5,6,7,8,9
6	7.98	Re_3_	C_48_H_82_O_19_	962.5444	1007.5423	[M + HCOO]^−^	0.20	961.5396, 799.4851, 637.4316, 475.3757	0,1,2,3,4,5,6,7,8,9
7	8.39	Noto R_4_	C_59_H_100_O_27_	1240.6446	1239.6300	[M − H]^−^	−5.49	1239.6337, 1107.5807,945.5306, 783.4845, 621.7329, 459.3895	1,2,3,4,5,6,7,8,9
8	8.42	Pseudo‐ginsenoside F_11_	C_42_H_72_O_14_	800.4922	799.4797	[M − H]^−^	−5.13	799.4790, 653.4228, 491.3741, 205.0721	0,1,2,3,4,5,6,7,8,9
9	9.13	Rg_2_	C_42_H_72_O_13_	784.4973	829.4946	[M + HCOO]^−^	0.36	783.4841, 637.4339, 475.3786	1,2,3,4,5,6,7,8,9
10	9.28	Rb_1_	C_54_H_92_O_23_	1108.6029	1107.5933	[M − H]^−^	−1.08	945.5416, 783.4899, 621.4372, 459.3840	0,1,2,3,4,5,6,7,8,9
11	9.44	Rh_1_	C_36_H_62_O_9_	638.4394	683.4367	[M + HCOO]^−^	0.44	475.3762, 391.2812, 161.0449, 101.0231	1,2,3,4,5,6,7,8,9
12	9.45	Malonyl Rb_1_	C_57_H_94_O_26_	1194.6033	1193.5935	[M − H]^−^	−1.17	1107.5955, 945.5403, 783.4929, 621.4344, 459.3835	0,1,2
13	9.46	20(R)‐Rg_2_	C_42_H_72_O_13_	784.4973	829.4946	[M + HCOO]^−^	0.36	783.4875, 637.4328, 475.3808, 391.2859	1,2,3,4,5,6,7,8,9
14	9.53	Rc	C_53_H_90_O_22_	1078.5924	1077.5824	[M − H]^−^	−1.48	945.5417, 783.4897, 621.4360, 459.3830	0,1,2,3,4,5,6,7,8,9
15	9.59	20 (R)‐Rh_1_	C_36_H_62_O_9_	638.4394	683.4367	[M + HCOO]^−^	0.44	475.3760, 391.2814, 161.0451, 101.0232	1,2,3,4,5,6,7,8,9
16	9.86	Ro	C_48_H_76_O_19_	956.4981	955.4881	[M − H]^−^	−1.67	793.4370, 613.3741, 569.3851, 455.3529	0,1,2,3,4,5,6,7,8,9
17	9.95	Rb_2_	C_53_H_90_O_22_	1078.5924	1077.5824	[M − H]^−^	−1.48	945.5421, 783.4897, 621.4352, 459.3811	0,1,2,3,4,5,6,7,8,9
18	10.11	Rb_3_	C_53_H_90_O_22_	1078.5924	1077.5835	[M − H]^−^	−0.46	945.5418, 783.4887, 621.4385, 459.3859	0,1,2,3,4,5,6,7,8,9
19	10.36	F_1_	C_36_H_62_O_9_	638.4394	683.4367	[M + HCOO]^−^	0.44	475.3768, 391.2845, 179.0547, 161.0442, 101.0229	1,2,3,4,5,6,7,8,9
20	10.46	Vinaginsenoside R_3_	C_48_H_82_O_17_	930.5546	975.5461	[M + HCOO]^−^	−6.36	929.5472, 767.4932, 605.4390	0,1,2,3,4,5
21	10.7	Chikusetsu‐saponin Iva	C_42_H_66_O_14_	794.4453	793.4375	[M − H]^−^	0.88	793.4377,631.3849,613.3724,569.3862,455.3529	4,5,6,7,8,9
22	10.86	Rd	C_48_H_82_O_18_	946.5501	945.5405	[M − H]^−^	−1.27	783.4882, 621.4388, 459.3857	0,1,2,3,4,5,6,7,8,9
23	10.87	Zingibroside R_1_	C_42_H_66_O_14_	794.4447	793.4375	[M − H]^−^	0.88	793.4364, 631.3820, 455.3544	0,1,2,3,4,5,6,7,8,9
24	10.97	Malonyl Rd	C_51_H_84_O_21_	1032.5505	1031.5421	[M − H]^−^	0.00	945.5405, 783.4887, 621.4318, 459.3823, 221.0659	0,1,2
25	11.39	Gypenoside XVII	C_48_H_82_O_18_	946.5496	945.5388	[M − H]^−^	−3.07	945.5531, 783.4888, 621.4363	1,2,3,4
26	12.06	Rg_6_	C_42_H_70_O_12_	766.4861	811.4827	[M + HCOO]^−^	−1.36	765.4747, 619.4252	1,2,3,4,5,6,7,8,9
27	12.22	Rg_4_	C_42_H_70_O_12_	766.4861	811.4827	[M + HCOO]^−^	−1.36	765.4718, 619.4199	1,2,3,4,5,6,7,8,9
28	12.87	20(S)‐Rg_3_	C_42_H_72_O_13_	784.4973	829.4946	[M + HCOO]^−^	0.36	783.4850, 621.4399, 459.3847	1,2,3,4,5,6,7,8,9
29	13.03	20(R)‐Rg_3_	C_42_H_72_O_13_	784.4973	829.4946	[M + HCOO]^−^	0.36	783.4847, 621.4335, 459.3839	1,2,3,4,5,6,7,8,9
30	13.42	20 (S)‐Rs_3_	C_44_H_74_O_14_	826.5079	871.5031	[M + HCOO]^−^	−2.07	783.4929, 621.4368, 459.3843, 375.2904	1,2,3,4,5,6,7,8,9
31	13.58	20(R)‐Rs_3_	C_44_H_74_O_14_	826.5079	871.5031	[M + HCOO]^−^	−2.07	783.4854, 621.4338, 459.3823, 375.2903	1,2,3,4,5,6,7,8,9
32	14.02	Rk_1_	C_42_H_70_O_12_	766.4867	811.4827	[M + HCOO]^−^	−1.36	765.4742, 603.4237	1,2,3,4,5,6,7,8,9
33	14.33	Rg_5_	C_42_H_70_O_12_	766.4867	811.4827	[M + HCOO]^−^	−1.36	765.4796, 603.4260	1,2,3,4,5,6,7,8,9
34	14.73	Rs_5_	C_44_H_72_O_13_	808.4967	853.4925	[M + HCOO]^−^	−2.11	765.4799, 603.4271	1,2,3,4,5,6,7,8,9
35	14.88	Rs_4_	C_44_H_72_O_13_	808.4967	853.4925	[M + HCOO]^−^	−2.11	765.4800, 603.4265	1,2,3,4,5,6,7,8,9

For PPT‐type ginsenosides, the compound was detected in negative ion mode, showing an acetate adduct ion at *m/z* 845.4897 [M + HCOO]^−^ and a deprotonated molecular ion at *m/z* 799.4824 [M − H]^−^. The tandem MS spectrum analysis revealed characteristic fragment ions at *m/z* 637.4305 and *m/z* 475.3785, as shown in Figure 2c. The fragmentation pathway demonstrated that the precursor ion *m/z* 799.4824 [M − H]^−^ first lost 1Glc to form *m/z* 637.4305, followed by the subsequent loss of 2Glc to yield the characteristic aglycone ion at *m/z* 475.3785. This fragmentation pattern was fully consistent with the literature data (Xü et al. 2000) for ginsenoside Rg_1_, thus confirming the identity of this compound as ginsenoside Rg_1_. The mass difference between the fragment ion at *m/z* 829.4946 and that at *m/z* 783.4841 is 46 Da, indicating that the ion at *m/z* 783.4841 is the quasi‐molecular ion peak. The secondary fragment ions at *m/z* 637.4339 and *m/z* 475.3786 can be observed, as shown in Figure 2d. Its fragmentation pattern shows that the ion at *m/z* 783.4841 successively loses 2Glc to generate the fragment ion at *m/z* 475.3786. Based on the comparison with literature data (Xü et al. 2000), this compound is identified as ginsenoside Rg_2_. The ion at *m/z* 475 is a characteristic ion of PPT‐type ginsenosides.

For OA‐type ginsenosides, the molecular ion peak of the compound is [M − H]^−^ at *m/z* 955.4897. An MS fragmentation analysis was performed on it, and the fragmentation pattern is as follows: The ion at *m/z* 955.4897 loses 1Glc, 2Glc and one molecule of H_2_O, and 2Glc, and one molecule of glucuronic acid (GlcA) respectively, generating secondary fragment ions at *m/z* 793.4370, 613.3741, and 455.3529. The peak at *m/z* 455.3529 is a characteristic ion peak of OA‐type saponins (Figure 2e). By comparing with the literature (Oh et al. 2024), this compound is identified as ginsenoside Ro.

For OT‐type ginsenosides, the molecular ion peak of the compound is [M − H]^−^ at *m*/*z* 799.4790, and its fragmentation pattern is as follows: The ion at *m/z* 799.4790 loses 1Rha to generate an ion at *m/z* 653.4228. Eventually, after losing 1Glc and 1Rha, the characteristic ion peak of OT‐type saponin at *m/z* 491.3741 is produced, as shown in Figure 2f. By comparing with the literature (Huang, Li, et al. 2019; Huang, Liu, et al. 2019) and the fragment ions, this compound is identified as pseudo‐ginsenoside F_11_.

### 3.2 Ginsenoside Dynamics and Transformation Mechanisms in AGTS0‐AGTS9

**FIGURE 3:**
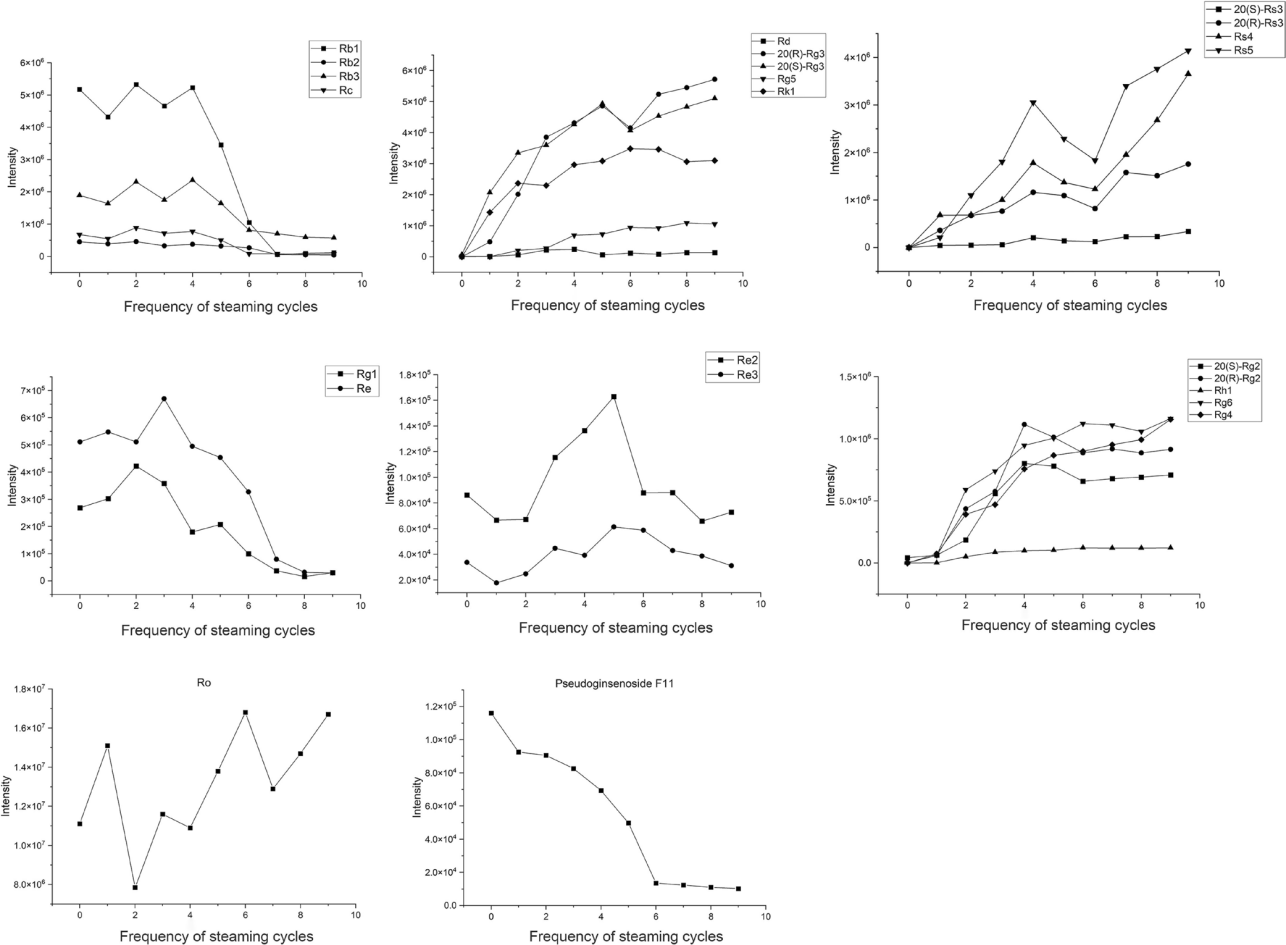
Peak intensity changes of representative ginsenosides in AGTS0‐AGTS9.

A significant variation in the types and contents of ginsenosides is observed between AGTS0 and groups AGTS1‐AGTS9 (Figure 1e). New saponin components such as ginsenosides Rk_1_, Rg_5_, Rh_1_, Rg_6_, and Rg_4_ are gradually detected in AGTS1‐AGTS9. With the increase in the number of steaming times, the peak intensities of these newly generated components gradually increase. The changes in the peak intensities of ginsenosides in AGTS0‐AGTS9 are shown in Figure 3. The results show that with the increase in the number of steaming times, the contents of proto‐ginsenosides, such as Rb_1_, Rb_2_, and Re continuously decrease, while the contents of rare ginsenosides such as Rg_3_, Rg_5_, Rk_1_, and Rg_2_ gradually increase. There are almost no proto‐ginsenosides left in AGTS7‐AGTS9, indicating that chemical transformations have occurred to the saponin components in American ginseng after steaming. Steaming is conducive to the transformation of proto‐ginsenosides into rare ginsenosides.

**FIGURE 4:**
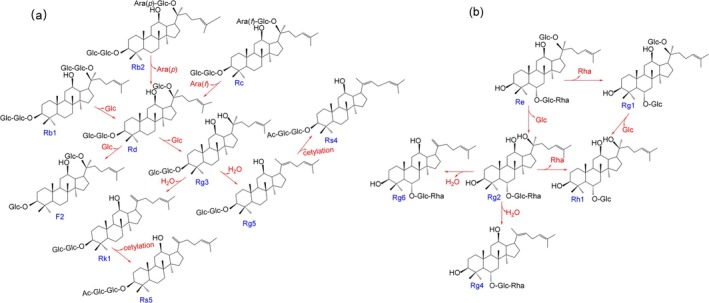
Structural changes of the main ginsenosides during the steaming and baking process of American ginseng: (a) Protopanaxadiol saponins. (b) Protopanaxatriol saponins.

During the steaming and baking processes, ginsenosides undergo significant structural transformations through various chemical reactions. Malonyl‐ginsenosides (malonyl‐Rb_1_, malonyl‐Rb_2_, malonyl‐Rg_1_, malonyl‐Re) exhibit intrinsic thermal instability. These acylated derivatives readily undergo decarboxylation under elevated temperatures, initially losing their malonyl moiety to form the corresponding neutral ginsenosides (Rb_1_, Rb_2_, Rg_1_, and Re). This thermal degradation pathway represents the primary transformation mechanism for malonyl‐ginsenosides during heat processing. For the PPD‐type ginsenosides shown in Figure 4a, the initial step involves the selective removal of sugar groups at the C‐20 position. Specifically, ginsenoside Rb_1_ loses a terminal glucosyl group, Rb_2_ loses a pyranose arabinosyl group, and Rc loses a furanose arabinosyl group, all resulting in the formation of ginsenoside Rd. As the thermal processing continues, Rd undergoes further transformation through two possible pathways: it can lose a glucosyl group either at the C‐20 position to form Rg3 or at the C‐3 position to produce F_2_. The resulting Rg_3_ then undergoes additional changes through hydrolysis to generate Rg_5_ and Rk_1_, which may subsequently undergo acetylation to form Rs_4_ and Rs_5_, respectively. For the PPT‐type ginsenosides illustrated in Figure 4b. Ginsenoside Re can lose either a rhamnose group at the C‐6 position to yield Rg_1_ or a glucose unit at the C‐20 position to form Rg_2_. The newly formed Rg_2_ can then follow different degradation pathways: it may lose its remaining rhamnose group at C‐6 to produce Rh_1_, or undergo hydrolysis to transform into Rg_6_ or Rg_4_. In conclusion, during the steaming and baking process, proto‐ginsenosides are mostly transformed into rare ginsenosides by breaking the glycosidic bonds and shedding glycosyl groups step by step. Meanwhile, reactions such as hydrolysis, isomerization, and acetylation also occur.

### 3.3 Neuroprotective Effects of AGTS0‐AGTS9 on Zebrafish Larvae

**FIGURE 5:**
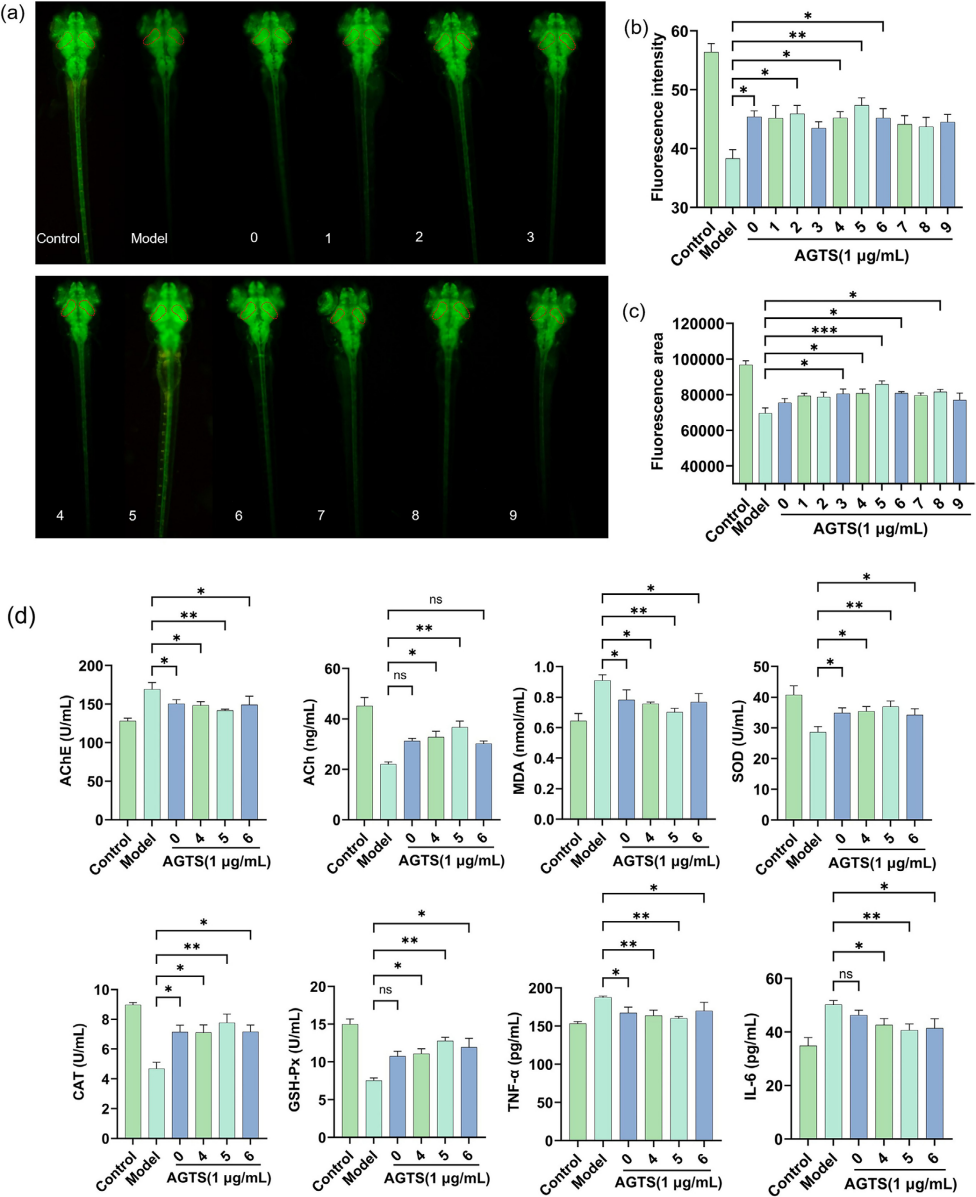
The neuroprotective effects of AGTS0‐AGTS9 samples on zebrafish: (a) Images of brain neurons of zebrafish. (b) Fluorescence area. (c) Fluorescence intensity. (d) The effects of AGTS on the levels of acetylcholine, the activity of acetylcholinesterase, oxidative stress markers, and inflammatory cytokines in the tissues of juvenile zebrafish. Results are presented as mean with SD (a b c, *n* = 15, d, *n* = 3). One‐way ANOVA with Dunnett's multiple comparison test: *p < 0.05, **p < 0.01, ***p < 0.001, compared with model group.

The fluorescence intensity is related to the number and activity of neurons, and the fluorescence area can reflect the number of neurons to a certain extent. Both of them can comprehensively and intuitively evaluate the neuroprotective activity of drugs. Figure 5a displays images of brain neurons from zebrafish in different groups. A notable decrease in neuron count was evident in the zebrafish brain exposed to 0.1 μg/mL AlCl_3_ compared to the untreated group, indicating neuronal damage. The administration of AGTS0‐AGTS9 ameliorated neuronal damage, leading to significant improvements in fluorescence intensity and area in zebrafish treated with AGTS4, AGTS5, and AGTS6 samples compared to the model group (*p* < 0.05 or *p* < 0.01). Notably, AGTS5 demonstrated the most effective improvement (*p* < 0.01). (Figure 5b,c).

In order to further verify the neuroprotective activities of AGTS4, AGTS5, and AGTS6, we analyzed the levels of neurotransmitters, oxidative stress, and inflammatory factors in the tissues of zebrafish larvae in the AGTS0, AGTS4, AGTS5, and AGTS6 groups. The results depicted in Figure 5d indicate a notable increase in the levels of AChE, MDA, TNF‐α, and IL‐6 in the tissues of zebrafish larvae within the model group, alongside a significant decrease in the activities of ACh, SOD, CAT, and GSH‐Px. The administration groups of AGTS0, AGTS4, AGTS5, and AGTS6 all decreased AChE, MDA, TNF‐α, and IL‐6 levels to varying extents and increased ACh, SOD, CAT, and GSH‐Px activities significantly (*p* < 0.05 or *P* < 0.01), suggesting a substantial enhancement in the neuroprotective properties of total saponin components in American ginseng following appropriate steaming, with AGTS5 demonstrating the highest neuroprotective activity (*p* < 0.01).

### 3.4 Behavioral Tasks of Adult Zebrafish

#### 3.4.1 NTT

**FIGURE 6:**
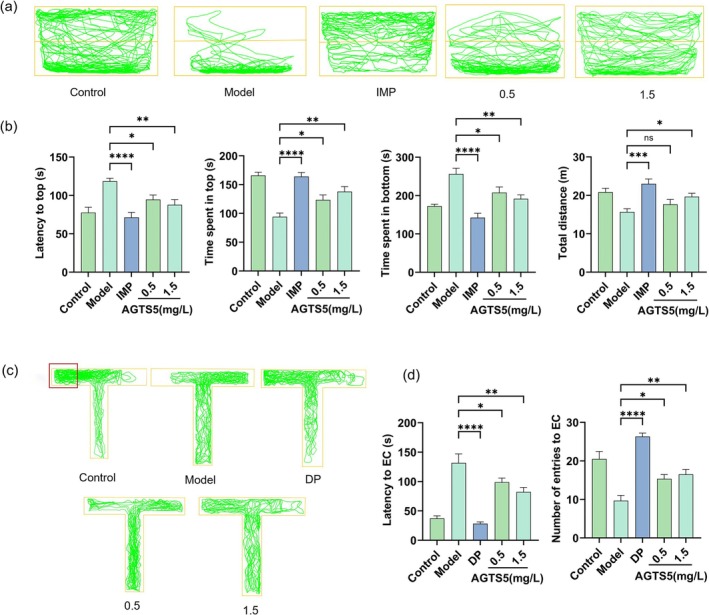
The impact of AGTS5 (AGTS5: 0.5, 1.5 mg/L) on the behavioral trajectories of adult zebrafish: (a) The representative movement trajectories of each group of zebrafish in the NTT. (b) Latency to top, time spent in top, time spent in bottom, total distance. (c) The representative movement trajectories of each group of zebrafish in the T‐maze. (d) Latency to the EC zone, number of entries to the EC zone. Results are presented as mean with SD (*n* = 6). One‐way ANOVA with Dunnett's multiple comparison test: *p < 0.05, **p < 0.01, ***p < 0.001, ****p < 0.0001, compared with model group.

From Figure 6a, the movement trajectories of zebrafish in the NTT can be observed. Administration of AlCl_3_ alone resulted in a significant increase in latency to reach the top and time spent at the bottom (Figure 6b), with reduced time spent at the top and total distance in the tank compared to the control group. Conversely, treatment with AGTS5, particularly at a dosage of 1.5 mg/L, significantly reduced latency to reach the top of the NTT (*p* < 0.01) and time spent at the bottom (*p* < 0.001). In addition, there was an increase in the time spent exploring at the top (*p* < 0.001) and the total distance traveled (*p* < 0.05) (Figure 6b). Moreover, IMP, conducted as a positive reference drug, displayed anxiolytic effects.

#### 3.4.2 T‐Maze Test

This study utilized a T‐maze test to assess the impact of AGTS5 on memory loss induced by AlCl_3_ in zebrafish. Figure 6c displays the travel pattern of zebrafish in the T‐maze. The latency to the EC zone in zebrafish treated with AlCl_3_ significantly increased compared to the control group. In addition, AlCl_3_ treatment also reduced the number of entries into the EC area in the T‐maze. In contrast, following the administration of AGTS5 at all doses, typically 1.5 mg/L, as well as the positive control medicine DP, there was a remarkable reduction in the latency to reach the EC zone (*p* < 0.01 or *p* < 0.001). Additionally, there was an apparent enhancement in the number of entries into the EC area (*p* < 0.01 or *p* < 0.001) (Figure 6d).

### 3.5 Nissl Staining

**FIGURE 7:**
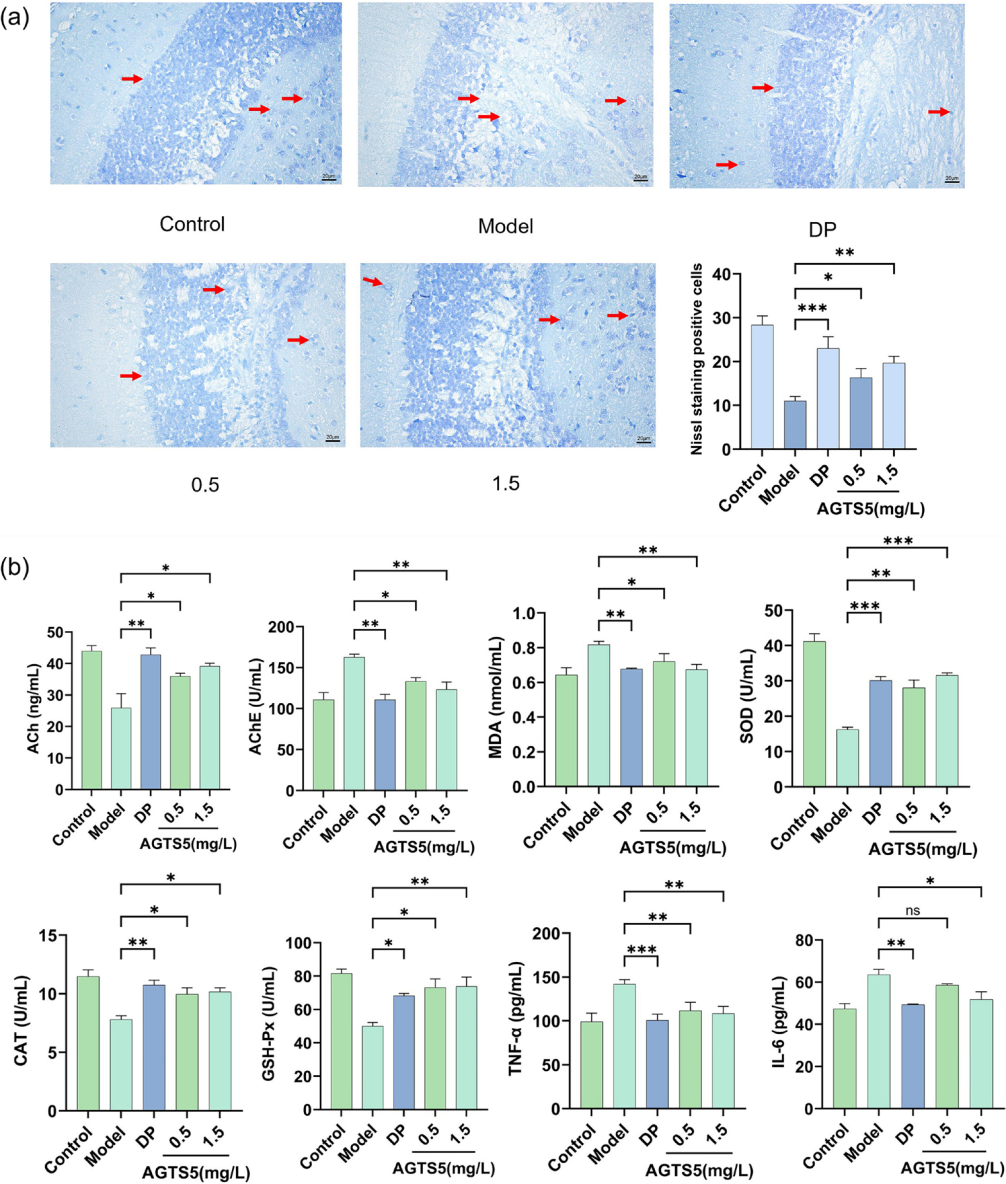
The effect of AGTS5 (AGTS5: 0.5, 1.5 mg/L) on Nissl bodies (×400) and biochemical analysis in the brains of adult zebrafish: (a) The effect of AGTS5 on Nissl bodies, the red arrow points to normal or damaged Nissl bodies. (b) The effects of AGTS5 on the levels of acetylcholine, the activity of acetylcholinesterase, oxidative stress markers and inflammatory cytokines. Results are presented as mean with SD (*n* = 3). One‐way ANOVA with Dunnett's multiple comparison test: *p < 0.05, **p < 0.01, ***p < 0.001, compared with model group.

The presence of Nissl bodies serves as an indicator of neuronal functional activity and metabolic vigor. When neurons sustain damage, Nissl bodies undergo dissolution, decrease, or even disappearance, thereby constituting a crucial marker for assessing neuronal cell injury. Figure 7a displays the Nissl staining results for the brains of zebrafish in different groups. In the control group, the neurons within the brains of zebrafish exhibited a dense and well‐organized arrangement, with abundant and plump Nissl bodies. In contrast, the brains of the group exposed to AlCl_3_ demonstrated a disordered arrangement of nerve cells, with enlarged intercellular spaces and a marked reduction in the number of Nissl bodies. Notably, after the administration of AGTS5, the nerve cells regained an orderly arrangement, and the number of Nissl bodies increased substantially (*p* < 0.05 or *p* < 0.01), demonstrating the neuroprotective efficacy of AGTS5 in mitigating AlCl_3_‐induced neuronal injury. This outcome underscores the potential of AGTS5 as a promising candidate for preserving neuronal integrity.

### 3.6 Biochemical Analysis

Figure 7b shows the levels of neurotransmitters, oxidative stress markers, and inflammatory factors in the brains of adult zebrafish. In the model group, the levels of AChE, MDA, TNF‐α, and IL‐6 significantly increased, whereas the activities of ACh, SOD, CAT, and GSH‐Px significantly decreased. After administration of AGTS5, the levels of AChE, MDA, TNF‐α, and IL‐6 were significantly reduced (*p* < 0.05 or *p* < 0.01), and the activities of ACh, SOD, CAT, and GSH‐Px were increased (*p* < 0.05 or *p* < 0.01), showing a dose‐dependent manner. These results indicate that AGTS5 exerts its effect in improving cognitive impairment by regulating neurotransmitter levels, enhancing antioxidant enzyme activities, and alleviating inflammation.

## 4 Discussion

This study systematically explored the dynamic changes of saponin components in American ginseng during steaming and their relationship with neuroprotective activity. In the initial stage of steaming, the samples of AGTS0‐AGTS3 mainly contained proto‐ginsenosides such as Rb_1_, Re, Rg_1_, and Rd, and also contained a small amount of rare ginsenosides such as Rg_2_, Rg_3_, Rg_5_, and Rk_1_. Therefore, they still possess certain neuroprotective activity. During the mid‐steaming phase, levels of proto‐ginsenosides showed a significant decrease, whereas the content of rare ginsenosides increased substantially. Research has demonstrated that rare ginsenosides exhibit low polarity and potent pharmacological activity (Wang et al. 2024; Xu et al. 2023). As a result, the AGTS4‐AGTS6 samples displayed markedly enhanced neuroprotective effects, with AGTS5 demonstrating the most pronounced activity. In the AGTS7‐AGTS9 samples, proto‐ginsenosides were nearly completely converted with continued accumulation of rare ginsenosides. Interestingly, the neuroprotective activity showed a relative decline during this stage, potentially due to the weaker bioactivity of secondary metabolites generated from excessive conversion of rare ginsenosides. This study reveals that the specific ratio of proto‐ginsenosides to rare ginsenosides in AGTS5 may be critical to its potent neuroprotective effects. Earlier spectrum‐effect relationship research by (Yang et al. 2025) indicated that the ethanol extract of American ginseng subjected to five cycles of steaming and baking exhibited significant neuroprotective activity. Its main bioactive components were characterized as the ginsenosides 20(S)‐Rg_3_, 20(R)‐Rg_3_, Rk_1_, and Rg_5_. Further comparative analysis of neuroprotective efficacy between four monomeric ginsenosides and the multiprocessed ethanol extract showed that the extract‐treated group had a higher nerve injury repair rate than groups receiving the four monomeric ginsenosides individually. While the four monomeric ginsenosides demonstrated notable neuroprotective effects, they could not fully replicate the efficacy of the ethanol extract. Substantial evidence indicates that different ginsenosides exert neuroprotection through distinct signaling pathways or targets, such as ginsenoside Rg_3_ reducing neurotoxicity by inhibiting proinflammatory factors (TNF‐α, IL‐1β, COX‐2) and suppressing activated microglia (Joo et al. 2008; Lee, Sur, et al. 2013; Lee, Park, et al. 2013). Rg_5_ mitigates neuroinflammation by regulating the Mitogen‐activated protein kinase (MAPK) and phosphatidylinositol 3‐kinase/protein kinase B (PI3K/Akt) pathways, thereby inhibiting the downstream transcription factors NF‐κB and AP‐1 (Lee, Sur, et al. 2013; Lee, Park, et al. 2013). Rg_2_ ameliorates Aβ_25–35_‐induced neurotoxicity and cognitive impairment by activating the PI3K/Akt signaling pathway (Cui et al. 2021). Re and Rd enhance neuroprotection by upregulating the expression of the choline acetyltransferase (ChAT)/vesicular acetylcholine transporter (VAChT) genes in Neuro‐2a cells (Kim et al. 2014). Therefore, we propose that the specific ratio between proto‐ginsenosides and rare ginsenosides in AGTS5 confers its potent neuroprotective activity, with rare ginsenosides playing a dominant role. In follow‐up studies, further in‐depth research can be conducted on the proportion of proto‐ginsenosides and rare ginsenosides in AGTS5 to determine the optimal ratio between the two for exerting neuroprotective effects. Steaming alters the proportion of ginsenosides in American ginseng, thereby influencing its bioactivity. We have only conducted a dynamic tracking of the neuroprotective activity. Whether AGTS5 also exhibits optimal efficacy in other pharmacological activities requires further research. For specific disease indications and health needs, by controlling the number of steaming and baking times, we can not only maximize the medicinal and edible value of American ginseng but also avoid poor curative effects and resource waste caused by excessive processing. The achievements of this study provide crucial scientific evidence for the standardized processing technology of American ginseng in the field of health foods.

Nissl bodies, primarily constituted by rough endoplasmic reticulum and free ribosomes, serve as pivotal sites for protein synthesis (Bhati et al. 2023). AChE can hydrolyze ACh and terminate the activation of cholinergic receptors by ACh, affecting nerve signal transduction and leading to cognitive impairment (Ferreira‐Vieira et al. 2016). MDA is a product of lipid peroxidation. An increase in its level reflects an elevated degree of oxidative stress in the body. SOD, GSH‐Px, and CAT can work synergistically to maintain the redox balance within cells and reduce the damage caused by oxidative stress to the nervous system (Santhi et al. 2025). Abnormal increases in the levels of TNF‐α and IL‐6 trigger a neuroinflammatory response, which is associated with various neurodegenerative diseases (Mantle and Lee 2018). Neuronal damage can lead to energy metabolism disorders, inflammation, oxidative stress, and so on. After administration of AGTS5, a significant increase was observed in both the fluorescence area and intensity of zebrafish neurons, accompanied by an elevation in the number of Nissl bodies within the brain. Additionally, the levels of AChE, MDA, TNF‐α, and IL‐6 in the tissues of zebrafish larvae and adults were significantly reduced, while the levels of ACh, SOD, GSH‐Px, and CAT were significantly increased. The findings suggest that AGTS5 can effectively reverse AlCl_3_‐induced neuronal damage, facilitating neuronal repair and regeneration. By enhancing energy supply for protein synthesis in the Nissl bodies of zebrafish brains, AGTS5 mitigates the adverse impact of oxidative stress and inflammatory cytokines on the structure and function of Nissl bodies, thereby exerting neuroprotective effects and enhancing learning and memory abilities. In conclusion, AGTS5 can exert neuroprotective effects and improve cognitive impairment through multiple targets and pathways, such as repairing damaged nerve cells, regulating neurotransmitters, alleviating oxidative stress, and reducing the levels of inflammatory factors. Studies have shown that ginsenoside Rb_1_ exerts a neuroprotective effect by regulating mitophagy and the NF‐κB pathway to inhibit the apoptosis of astrocytes (Ni et al. 2022). Rg_1_ inhibits the expression of cyclin‐dependent kinase 5 (CDK5) and the phosphorylation of peroxisome proliferator‐activated receptor γ (PPARγ), and then downregulates the target gene BACE1 of PPARγ to reduce the deposition of amyloid‐β (Aβ) (Quan et al. 2020). Rg_5_ can inhibit the activity of acetylcholinesterase in a dose‐dependent manner, significantly improving the memory impairment in mice induced by scopolamine (Kim et al. 2013). Pseudoginsenoside F_11_ protects the synaptic structure by inhibiting the production of APP and Aβ, restoring the activities of SOD and GSH‐Px, reducing the content of MDA, and regulating the expression of tau phosphorylation (Wang et al. 2013). The AGTS5 extracted in this study is rich in various ginsenosides and has multiple aspects of its action mechanism. This suggests that there may be a synergistic effect among the ginsenosides in AGTS5, which enables it to achieve a protective effect on the nervous system through multiple targets and pathways.

AGTS5 contains abundant ginsenosides, especially rare ginsenosides, and demonstrates remarkable superiority in neuroprotective activity. Moreover, AGTS5 acts through a multitarget, multipathway mechanism. Ginsenosides are the main active components for neuroprotection and have no severe adverse reactions (Christensen 2009). Studies have shown that ginsenosides can protect nerve cells from the harm of neurodegenerative diseases by reducing the deposition of Aβ, inhibiting the phosphorylation of tau protein, alleviating neuroinflammation, combating oxidative stress, promoting neurite outgrowth, and regulating the levels of neurotransmitters (Huang, Li, et al. 2019; Huang, Liu, et al. 2019; Radad et al. 2011). Therefore, AGTS5 has great potential in the development of natural drugs for neurodegenerative diseases. Compared with monomeric ginsenosides, especially the high preparation costs of rare monomeric ginsenosides such as Rg_3_ and Rg_5_, AGTS5 can be obtained in batches through traditional steaming processes, which significantly reduces the raw material costs and contributes to the development of functional foods for cognitive impairment. Clinically, the use of AGTS5 allows for further exploration of its indications, which is beneficial for delaying the symptoms of patients with neurodegenerative diseases and improving their quality of life. However, the clinical application of AGTS5 still faces multiple challenges. Its composition is complex, and the mechanism of the synergistic effect among different monomeric saponins is not yet fully understood. Currently, there is relatively little research related to AGTS5, and further verification of its safety and effectiveness is required for clinical translation. In addition, the blood–brain barrier (BBB), as the primary protective mechanism of the central nervous system, restricts the delivery efficiency of ginsenosides to the brain. Ginsenosides Rb_1_, Rg_1_, Ro, and Re can be detected in the brain after oral administration, indicating that they can cross the BBB. However, the concentrations of these ginsenosides in the brain are 8–15 times lower than those of the corresponding components in the plasma (An et al. 2021; Wang et al. 2010). It is worth noting that the rapid development of nanotechnology provides a solution to this problem. Yuwen Ting used nanotechnology to emulsify adlay bran oil (ABO), which significantly improved the bioavailability of ABO (Ting et al. 2019). After ginsenoside Rg_3_ is encapsulated by poly (lactic‐*co*‐glycolic acid) (PLGA) nanoparticles, its permeability through the BBB increases (Aalinkeel et al. 2018). Therefore, in the future, it is feasible to attempt to optimize the nervous system targeting ability of AGTS5 through nano delivery technology, thereby enhancing its clinical application value.

## 5 Conclusion

This study systematically explored the effects of the steaming process on the saponin components of American ginseng and their neuroprotective activities. Our findings demonstrate that the steaming process can effectively promote the conversion of proto‐ginsenosides into rare ginsenosides. Its molecular mechanism mainly involves the selective cleavage of glycosidic bonds, accompanied by a series of structural modification reactions such as hydrolysis and acetylation. More notably, moderate steaming (AGTS5) can maximize the neuroprotective activity of the saponin components in American ginseng. AGTS5 can exhibit excellent neuroprotective and cognitive‐enhancing activities by regulating the levels of neurotransmitters, oxidative stress markers, and inflammatory factors. This study can not only provide a reference for the processing standards of American ginseng in the nutritional and health products industry, but also contribute to the development of multitarget neuroprotectants based on natural products and functional foods for improving cognitive impairment. In the future, AGTS5 can be added to functional foods such as functional yogurt, nutritious pastries, and herbal health teas, facilitating the in‐depth transformation and application of natural product resources in the field of neurological health.

## Author Contributions

**Yuting Yang:** writing – original draft (lead). **Shuyun Liang:** writing – original draft (equal). **Mengdan Xu:** writing – original draft (supporting). **Xiaokang Liu:** writing – original draft (supporting). **Liru Zhao:** funding acquisition (lead). **Guangzhi Cai:** supervision (lead), writing – review and editing (lead). **Yunlong Guo:** date curation (supporting). **Jiyu Gong:** methodology (supporting).

## Conflicts of Interest

The authors declare no conflicts of interest.

## Data Availability

The data supporting the findings of this study are available upon request from the corresponding author.
